# Growth-stimulatory activity of TIMP-2 is mediated through c-Src activation followed by activation of FAK, PI3-kinase/AKT, and ERK1/2 independent of MMP inhibition in lung adenocarcinoma cells

**DOI:** 10.18632/oncotarget.5466

**Published:** 2015-11-07

**Authors:** Han Ie Kim, Hyun-Sung Lee, Tae Hyun Kim, Ju-Seog Lee, Seung-Taek Lee, Seo-Jin Lee

**Affiliations:** ^1^ Department of Life Science & Biotechnology, Shingyeong University, Gyeonggi-do, 445-741, Republic of Korea; ^2^ Division of Thoracic Surgery, Michael E. DeBakey Department of Surgery, Baylor College of Medicine, Houston, TX, 77030, U.S.A; ^3^ Department of Systems Biology, Division of Cancer Medicine, The University of Texas MD Anderson Cancer Center, Houston, TX, 77054, U.S.A; ^4^ Department of Biochemistry, College of Life Science and Biotechnology, Yonsei University, Seoul, 120-749, Republic of Korea

**Keywords:** TIMPs, tumorigenesis, c-Src, lung adenocarcinoma, PI3-kinase/AKT pathway

## Abstract

Tissue inhibitors of metalloproteinases (TIMPs) control extracellular matrix (ECM) homeostasis by inhibiting the activity of matrix metalloproteinases (MMPs), which are associated with ECM turnover. Recent studies have revealed that TIMPs are implicated in tumorigenesis in both MMP-dependent and MMP-independent manners. We examined a mechanism by which TIMP-2 stimulated lung adenocarcinoma cell proliferation, independent of MMP inhibition. The stimulation of growth by TIMP-2 in A549 cells required c-Src kinase activation. c-Src kinase activity, induced by TIMP-2, concomitantly increased FAK, phosphoinositide 3-kinase (PI3-kinase)/AKT, and ERK1/2 activation. Selective knockdown of integrin α3β1, known as a TIMP-2 receptor, did not significantly change TIMP-2 growth promoting activity. Furthermore, we showed that high TIMP-2 expression in lung adenocarcinomas is associated with a worse prognosis from multiple cohorts, especially for stage I lung adenocarcinoma. Through integrated analysis of The Cancer Genome Atlas data, TIMP-2 expression was significantly associated with the alteration of driving genes, c-Src activation, and PI3-kinase/AKT pathway activation. Taken together, our results demonstrate that TIMP-2 stimulates lung adenocarcinoma cell proliferation through c-Src, FAK, PI3-kinase/AKT, and ERK1/2 pathway activation in an MMP-independent manner.

## INTRODUCTION

The hallmarks of cancer cells include sustained proliferative signaling, evading growth suppressors, resisting cell death, enabling replicative immortality, inducing angiogenesis, activating invasion and metastasis, reprogramming energy metabolism, and evading immune destruction [1, 2]. As tumors progress toward metastasis, the initial proteolytic remodeling of the extracellular matrix (ECM) is essential, especially by matrix metalloproteinases (MMPs), a family of zinc-dependent endopeptidases that are produced by tumor cells and stromal cells [3]. Tissue inhibitors of metalloproteinases (TIMPs) are a family of four proteins, TIMP-1 through TIMP-4, that act as endogenous inhibitors of MMPs [4]. Balanced interaction between MMPs and TIMPs regulates ECM homeostasis during physiological conditions, whereas an imbalance in MMP and TIMP expression and/or activity is present in pathological conditions such as arthritis, cardiovascular disease, emphysema, retinopathies, and cancer [5].

Clinical studies show that elevated MMP expression in human cancers generally increases with tumor progression [6]. Paradoxically, overexpression of TIMP-1 and/or TIMP-2 often positively correlates with tumorigenesis in breast, colorectal, lung, and gastric cancer patients [7–12]. Recent reports have discovered a role for TIMPs that is independent of MMP inhibition and therefore supports the paradoxical findings described above. It has been widely shown that TIMPs stimulate cell proliferation and inhibit apoptosis in various types of cancer cells. TIMP-1 was reported to stimulate human osteosarcoma MG-63 cell, Raji lymphoma cell, and UT7 leukemia cell proliferation. [13–17]. TIMP-1 inhibits apoptosis in human breast carcinoma T-47D cells [18]. TIMP-2 has been shown to stimulate A549 lung adenocarcinoma cell, HT1080 fibrosarcoma cell, MG-63 osteosarcoma cell, and A2058 melanoma cell proliferation [14, 15, 19, 20]. TIMP-4 reportedly stimulates MDA-MB-231 breast cancer cell growth [21] and decreases apoptosis in MDA-MB-435 derived tumors [22].

All of these studies show that TIMPs stimulate proliferation in a wide range of cancer cells. There are several mechanisms that are associated with the growth-stimulatory activity of TIMPs. TIMP-1 increases cell proliferation through the activation of p38 and JNK1/2 in UT7 leukemia cells and through the activation of the extracellular signal-regulated kinase (ERK) and p38 pathways in MDA-MB-435 cells [14, 16]. TIMP-2 mediates a mitogenic response by stimulating adenylate cyclase to produce cyclic AMP (cAMP), which in turn activates protein kinase A (PKA) in HT-1080 cells [20]. In MG-63 cells, TIMP-2 stimulates cell growth via the PKA-Ras-phosphoinositide 3-kinase (PI3-kinase) signaling pathway [14]. In addition, TIMP-2 promotes cell growth by activating nuclear factor kappa B (NF-κB) in A549 cells and melanoma cells [19, 23]. In human foreskin HSF4 fibroblasts, the growth-stimulatory activity of TIMP-2 is known to require insulin [24]. In contrast, Lizarraga et al. demonstrated that TIMP-2 mediates cell growth in the absence of insulin in A549 cells [19].

Recently, TIMP-2 overexpression was detected in the lungs of mice with Lewis lung carcinoma and was positively correlated with tumor progression in non-small cell lung carcinoma (NSCLC) [12, 25]. Previous findings from *in vitro* and clinical studies support the idea that TIMP-2′s growth-stimulatory activity may play a key role in lung tumorigenesis. Thus, we examined the signaling pathways by which TIMP-2 stimulates cell proliferation in lung adenocarcinoma cells. Additionally, we performed a genome-wide survey of gene-expression data to evaluate the association of TIMP-2's growth-stimulatory activity with lung adenocarcinoma prognosis in multiple independent cohorts. We also tested the correlation between TIMP-2 and the alteration of driving genes through integrated analysis of The Cancer of Genome Atlas (TCGA) for lung adenocarcinoma.

## RESULTS

### TIMP-2 stimulated proliferation of lung adenocarcinoma cell lines in an MMP-independent manner

**Figure 1:**
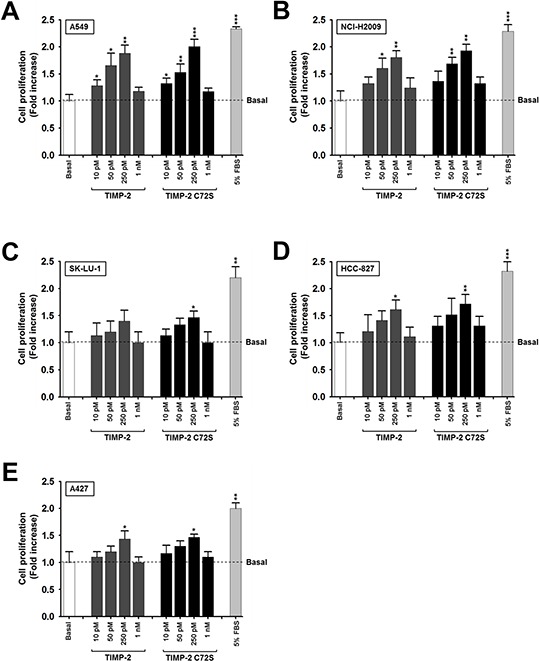
Effect of TIMP-2 or TIMP-2 C72S on the proliferation of several lung adenocarcinoma cell lines We used A549 **A.** NCI-H2009 **B.** SK-LU-1 **C.** HCC-827 **D.** and A427 **E.** cells to perform BrdU incorporation assays. Lung adenocarcimoma cell lines were serum-starved in the presence of various concentrations of TIMP-2 or TIMP-2 C72S for 48 hr and then BrdU incorporation was evaluated. Standard deviations were calculated from experiments performed in triplicate in three independent assays. Statistical significance is indicated. *p < 0.05 **p < 0.01 ***p < 0.001 when compared with untreated cells.

In previous reports, TIMP-2 stimulated A549 lung adenocarcinoma cell proliferation at concentrations of 10–50 pM [19, 24]. To further clarify the relationship between TIMP-2 concentration and growth stimulation, various concentrations of TIMP-2 were tested for their ability to stimulate BrdU incorporation in several lung adenocarcinoma cell lines, including A549, NCI-H2009, SK-LU-1, HCC-827, and A427. To exclude the effect of MMP inhibition, a TIMP-2 C72S mutant that cannot inhibit MMP activity, was included in all of the experiments with TIMP-2. The highest levels of proliferation were achieved when the cells were treated with 250 pM of either TIMP-2 or TIMP-2 C72S. TIMP-2 had the greatest effect on A549 and NCI-H2009 cell proliferation. TIMP-2 treatment increased A549 cell proliferation 1.9-fold over the basal proliferation level without TIMP-2 treatment. TIMP-2 C72S treatment increased A549 cell proliferation 2-fold over the basal level (Figure 1A). Similarly, in NCI-H2009 cells, TIMP-2 increased the proliferation rate 1.8-fold over the basal level and TIMP-2 C72S increased the proliferation rate 1.9-fold over the basal level (Figure 1B). Fetal bovine serum (5% FBS) was used as a positive control and stimulated a 2.3-fold increase in proliferation over the basal proliferation levels in both cell lines (Figure 1A and 1B). Treating the other lung adenocarcinoma cell lines with 250 pM of either TIMP-2 or TIMP-2 C72S stimulated 1.4-fold to 1.7-fold increases in cell proliferation in a statistically significant fashion (*p* < 0.05) when compared with untreated cells (Figure 1C–1E). This data demonstrates that TIMP-2 efficiently stimulated proliferation in several lung adenocarcinoma cell lines in an MMP-independent manner. The most pronounced effects on proliferation were detected in A549 and NCI-H2009 cells. Therefore, we utilized A549 cells in experiments to identify the mechanism by which TIMP-2 stimulates cell proliferation, and we used NCI-H2009 cells to confirm our results from A549 cells.

### TIMP-2 activates ERKs, PI3-kinase, NF-κB, and the Src family of kinases in insulin-independent manner

**Figure 2:**
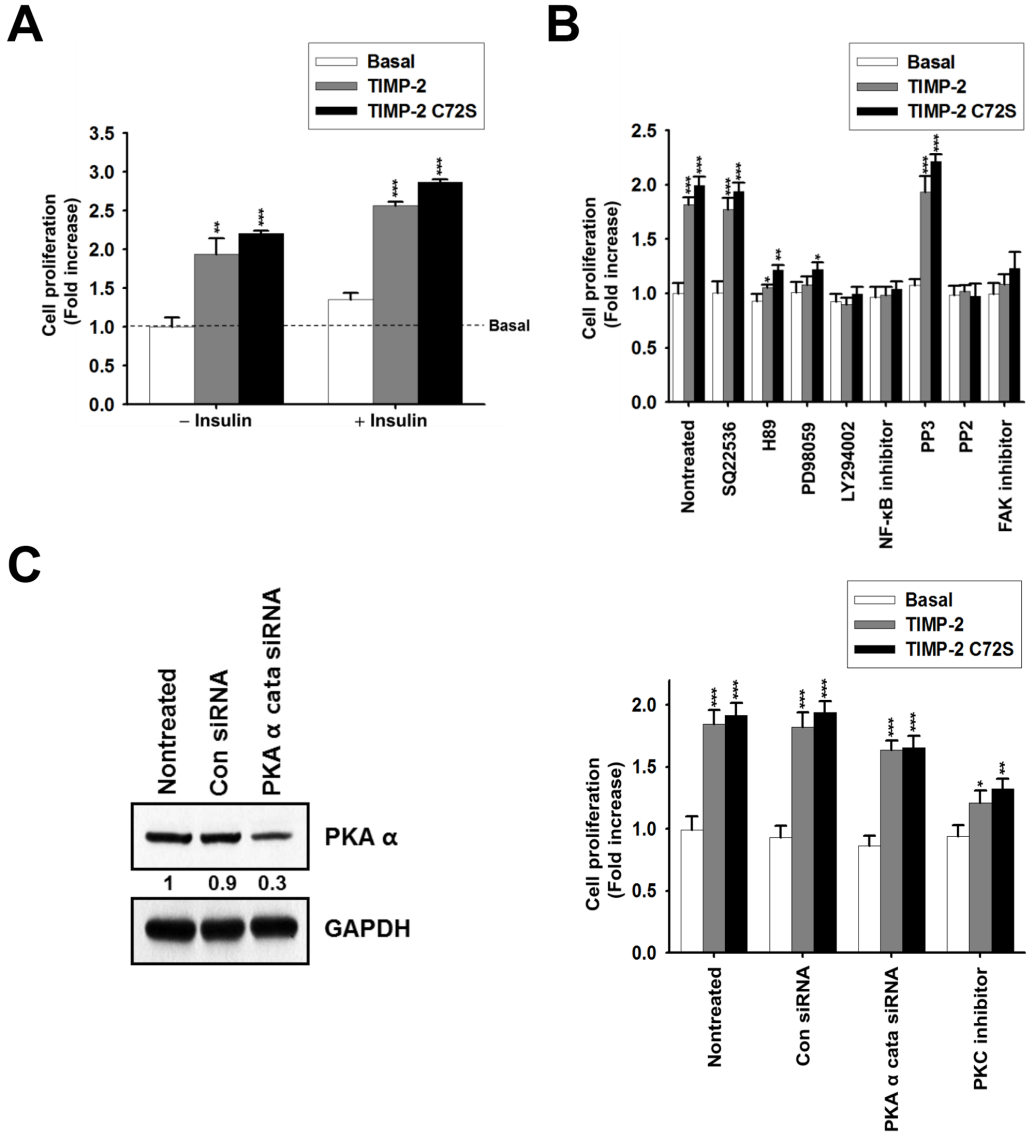
Effect of insulin and signaling inhibitors on TIMP-2 or TIMP-2 C72S-induced A549 cell proliferation **A.** BrdU incorporation assay in serum-starved A549 cells treated with 250 pM of either TIMP-2 or TIMP-2 C72S in the absence of and presence of insulin (5 μg/ml) for 48 hr. **B.** BrdU incorporation assay in serum-starved A549 cells incubated for 48 hr in DMEM containing 250 pM of either TIMP-2 or TIMP-2 C72S with or without various signaling inhibitors for 30 min. **C.** Western blot to evaluate PKAα catalytic subunit and GAPDH expression in A549 cells transfected with control siRNA (Con siRNA) or PKAα cata siRNA (left panel). BrdU incorporation assay in Con siRNA and PKAα cata siRNA-transfected A549 cells treated with 250 pM of either TIMP-2 or TIMP-2 C72S in serum-free medium for 48 hr with or without 100 nM PKC inhibitor (right panel). Standard deviations were calculated from experiments performed in triplicate in three independent assays. Statistical significance is indicated. *p < 0.05 **p < 0.01 ***p < 0.001 when compared with untreated cells with TIMP-2 or TIMP-2 C72S.

The growth-stimulatory activity of TIMP-2 requires insulin in human foreskin fibroblasts but does not require insulin in A549 cells [19, 24]. To evaluate the effect of insulin on TIMP-2-induced cell proliferation in an MMP-independent manner, we performed cell proliferation assays using the TIMP-2 C72S mutant. Insulin treatment increased basal cell proliferation by ~1.2-fold compared with the basal proliferation level of cells that did not receive insulin treatment; however, TIMP-2 and TIMP-2 C72S treatment increased cell proliferation to similar levels irrespective of insulin treatment (Figure 2A). This finding suggests that TIMP-2 induces A549 cell proliferation in an insulin-independent and a MMP-independent manner.

To determine the signaling pathways involved in the growth-stimulatory activity of TIMP-2, we tested the effects of various inhibitors on TIMP-2-induced cell proliferation. The inhibitors used were as follows: SQ22536, an inhibitor of adenylate cyclase; H89, an inhibitor of PKA; PD98059, an inhibitor of mitogen-activated protein kinase kinase (MEK); LY294002, an inhibitor of PI 3-kinase; NF-κB inhibitor; PP2, an inhibitor of the Src family of kinases; PP3, a negative control for PP2; focal adhesion kinase (FAK) inhibitor I; and Gö 6976, an inhibitor of PKC. In previous reports, tyrosine kinase inhibitors, such as genistein, erbstatin, or herbimycin A, significantly blocked TIMP-2-mediated cell growth [26]. Because the Src family of tyrosine kinases plays a key role in cell growth, we included PP2 in our assay [27]. TIMP-2-induced cell proliferation was significantly inhibited by H89, PD98059, LY294002, NF-κB inhibitor, PP2, and FAK inhibitor treatment, but was not inhibited the adenylate cyclase inhibitor, SQ22536 (Figure 2B). This result suggests that TIMP-2 growth-stimulatory activity in A549 cells involves the activation of ERKs, PI 3-kinase, and NF-κB, which is consistent with previous results [19]. H89 inhibits PKA as well as other signaling molecules, such as casein kinase I, myosin light chain kinase (MLCK), protein kinase C (PKC), and ROCK-II, suggesting that TIMP-2 growth-stimulatory activity may involve other signaling molecules in addition to cAMP/PKA. To confirm this, we assessed the effect of TIMP-2 on cell proliferation after inhibiting PKA activity using siRNA against the PKAα catalytic subunit (PKAα cata siRNA) or by inhibiting PKC activity using Gö 6976 (PKC inhibitor). Transfection of PKAα cata siRNA into A549 cells reduced PKAα catalytic subunit protein expression by ~70% compared with control siRNA treated (Con siRNA) cells (Figure 2C). In PKAα cata siRNA-transfected A549 cells, both TIMP-2 and TIMP-2 C72S stimulated cell proliferation to levels that were comparable to those of the control siRNA-transfected cells. In A549 cells treated with PKC inhibitor, TIMP-2 and TIMP-2 C72S-induced cell proliferation levels were significantly reduced compared with the respective proliferation levels in non-treated cells (Figure 2C). This result suggests that TIMP-2 growth-stimulatory activity may involve PKC via a cAMP/PKA independent mechanism.

### TIMP-2 growth-stimulatory activity is mediated through c-Src activation

**Figure 3:**
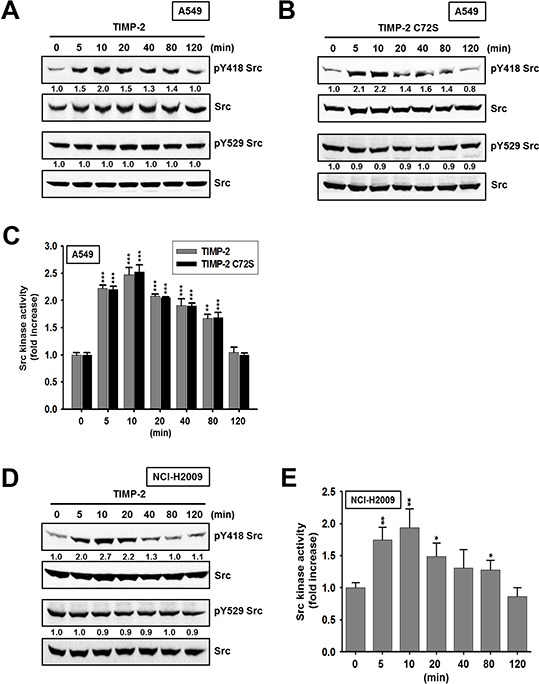
Activation of c-Src kinase activity by TIMP-2 or TIMP-2 C72S A549 cells treated with 250 pM of either TIMP-2 **A.** or TIMP-2 C72S **B.** and NCI-H2009 cells treated with 250 pM TIMP-2 **D.** for indicated time periods were subjected to western blot analysis with antibodies recognizing pY418 Src, pY529 Src, and Src. Western blots shown are representative of three independent experiments. Relative band intensities were measured using Adobe photoshop software and integrated densities were normalized against the Src loading control. A549 cells **C.** or NCI-H2009 cells **E.** treated with 250 pM of either TIMP-2 or TIMP-2 C72S for indicated time periods were harvested. The c-Src kinase activites of the cell lysates were measured as described in “Materials and methods”. Standard deviations were calculated from experiments performed in triplicate in three independent assays. Statistical significance is indicated. *p < 0.05 **p < 0.01 ***p < 0.001 when compared with treated cells with TIMP-2 or TIMP-2 C72S (0 min).

PP2 strongly inhibited the TIMP-2-induced cell proliferation (Figure 2B), indicating that the Src family of tyrosine kinases is involved in TIMP-2 growth-stimulatory activity. We hypothesized that if c-Src is involved in TIMP-2 growth-stimulatory activity, then FAK should be activated by c-Src. Therefore, we evaluated TIMP-2-induced cell proliferation after treatment with a FAK inhibitor. TIMP-2 did not induce cell proliferation in the presence of FAK inhibitor (Figure 2B). This result supports the hypothesis that c-Src may be activated by TIMP-2. This prompted us to analyze whether TIMP-2 activates c-Src in A549 cells. We determined the level of c-Src activation after treating cells with TIMP-2 by performing western blots to evaluate the phosphorylation status of c-Src on either Y418, which is essential for maximal c-Src catalytic activity, or Y529. Both TIMP-2 and TIMP-2 C72S induced phosphorylation of Src at Y418, with the highest level of phosphorylation detected after 10 min of treatment (Figure 3A). In contrast, TIMP-2 and TIMP-2 C72S treatment did not affect Src phosphorylation at Y529 (Figure 3B). To confirm activation of c-Src by TIMP-2, c-Src kinase activity was evaluated. We found that c-Src kinase activity increased when the cells were treated with TIMP-2 or TIMP-2 C72S. Maximal Src kinase activation was approximately 2.5-fold higher than control levels after 10 min of TIMP-2 or TIMP-2 C72S treatment (Figure 3C). To confirm these results from A549 cells, we evaluated c-Src kinase phosphorylation and activity in NCI-H2009 cells treated with TIMP-2 (Figure 3D and 3E). The results from NCI-H2009 cells were consistent with those from A549 cells. Our findings demonstrate that TIMP-2 significantly induces c-Src kinase activity in an MMP-independent manner.

**Figure 4:**
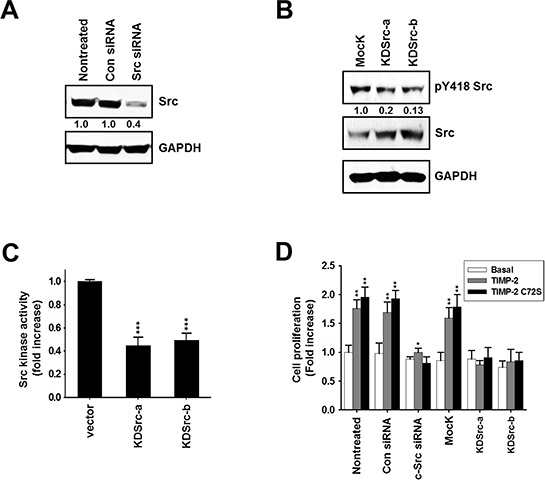
Effect of TIMP-2 or TIMP-2 C72S on growth-stimulatory activity in c-Src siRNA-transfected cells and in kinase-dead Src (K297R) A549 cell lines **A.** A549 cells were transfected with either control siRNA or c-Src siRNA. After 48 hr, the cells were harvested and the cell lysates were subjected to western blot analysis with antibodies that detect c-Src and GAPDH. **B.** Two kinase-dead Src (K297R) expressing A549 cell lines (KDSrc-a and KDSrc-b) were cultured and harvested. The cell lysates were subjected to western blot analysis with antibodies that detect pY418 Src, c-Src, and GAPDH. **C.** The KDSrc-a and KDSrc-b cells were harvested and c-Src kinase activity in the cell lysates was measured **D.** 250 pM TIMP-2 or TIMP-2 C72S was added to c-Src siRNA-transfected cells and the KDSrc-a and KDSrc-b A549 cells starved in serum-free medium. After 48 hr, cell proliferation was measured by BrdU incorporation assay. Standard deviations were calculated from experiments performed in triplicate in three independent assays. Relative band intensities were measured by adobe photoshop software and integrated densities were normalized against the Src loading control. Statistical significance is indicated. *p < 0.05 **p < 0.01 ***p < 0.001 when compared with untreated cells with TIMP-2 or TIMP-2 C72S.

To further verify that c-Src is involved in the growth-stimulatory activity of TIMP-2, we performed experiments using c-Src-silenced A549 cells and A549 stable cell lines expressing kinase-dead Src (K297R). Transfecting c-Src siRNA into A549 cells reduced c-Src protein expression by ~80% (Figure 4A). Two stable clones expressing kinase-dead Src (K297R), KDSrc-a and KDSrc-b, showed 2.5-fold and 3.0-fold higher c-Src expression than the vector-transfected cells, respectively. However, KDSrc-a had an 80% reduction in Src phosphorylation at Y418 and KDSrc-b had a 87% reduction in Src phosphorylation at Y418 compared with that in the control cells (Figure 4B). Accordingly, Src kinase activity was reduced by 55% in the KDSrc-a cell line and 50% in the KDSrc-b cell line compared with the level of Src activity in the control cells (Figure 4C). TIMP-2 and TIMP-2 C72S did not stimulate cell proliferation in either the c-Src siRNA A549 cells or the kinase-dead cell lines (KDSrc-a and KDSrc-b; Figure 4D). These findings support the idea that TIMP-2 activates c-Src to promote cell proliferation in A549 cells.

### TIMP-2 growth-stimulatory activity is mediated through c-Src activation followed by FAK, PI3-kinase/AKT, and ERK1/2 pathway activation in an MMP-independent manner in A549 cells

**Figure 5:**
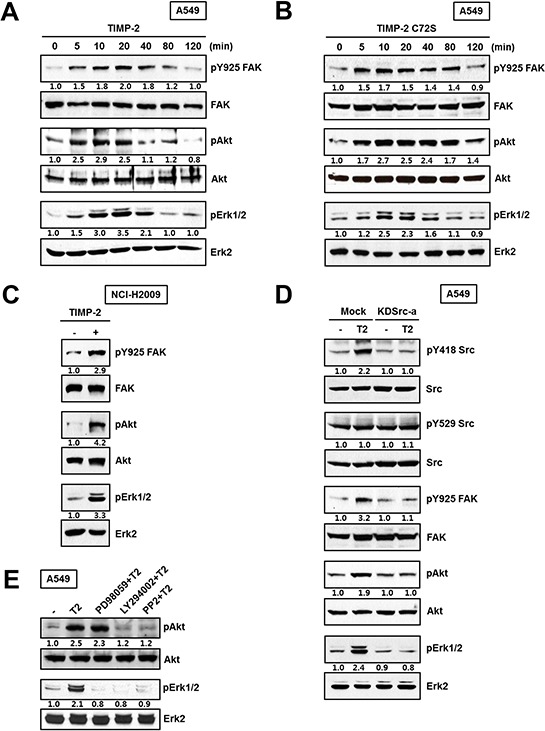
Effect of TIMP-2 or TIMP-2 C72S on the activation of downstream signaling proteins A549 cells were treated with 250 pM of either TIMP-2 **A.** or TIMP-2 C72S **B.** for indicated time periods. **C.** NCI-H2009 cells were treated with 250 pM TIMP-2 for 10 min. **D.** TIMP-2 was treated for 10 min in Mock and KDSrc-a A549 cells. The cell lysates were subjected to western blot analysis with antibodies that recognized pY925 FAK, FAK, pS473Akt, Akt, pERK1/2, and ERK2. Western blots shown are representative of three independent experiments. **E.** Serum-starved A549 cells were pretreated with the indicated signaling inhibitors for 30 min prior to TIMP-2 treatment. After 10 min, the cells were harvested and the cell lysates were subjected to Western blotting for pS473Akt, Akt, pERK1/2, and ERK2. Relative band intensities were measured using Adobe photoshop software and integrated densities were normalized against the FAK, Akt, or ERK2 loading control.

The SH2 domain of c-Src binds to FAK when FAK is phosphorylated at Y397. In turn, c-Src kinase activity contributes to maximal FAK activation by phosphorylating FAK at Y925 [28]. To examine the involvement of FAK, MAP kinase, or PI3-kinase during TIMP-2-induced A549 cell proliferation, we evaluated the following: FAK phosphorylation on Y925 downstream of c-Src, AKT phosphorylation downstream of PI3-kinase, and ERK1/2 phosphorylation. Both TIMP-2 and TIMP-2 C72S increased FAK phosphorylation on Y925, AKT phosphorylation, and ERK1/2 phosphorylation (Figure 5A and 5B). In NCI-H2009 cells, TIMP-2 induced the phosphorylation of those same proteins (Figure 5C). Next, we evaluated signaling protein activation in KDSrc-a cells, the A549 stable cell line expressing kinase-dead Src (K297R). In KDSrc-a cells, TIMP-2 treatment did not induce c-Src, FAK, ERK1/2, or AKT phosphorylation when compared with that in vector-transfected cells (Figure 5D). This result suggests that FAK, ERK1/2, PI3-kinase, and AKT are downstream of c-Src. To further analyze the order of these downstream signaling proteins, we evaluated the effect of TIMP-2 treatment in the absence or presence of the following kinase inhibitors: a MEK inhibitor (PD98059), a PI3-kinase inhibitor (LY294002), and a Src inhibitor (PP2). Pretreating the cells with PI3-kinase inhibitor completely suppressed TIMP-2-induced ERK1/2 phosphorylation, whereas the MEK inhibitor did not affect TIMP-2-induced AKT phosphorylation (Figure 5E). This result suggests that PI3-kinase is upstream of ERK1/2. Taken together, our data suggest that TIMP-2 growth-stimulatory activity is mediated by c-Src followed by the FAK, PI3-kinase/AKT, and ERK1/2 pathways in an MMP-independent manner in A549 cells.

**Figure 6:**
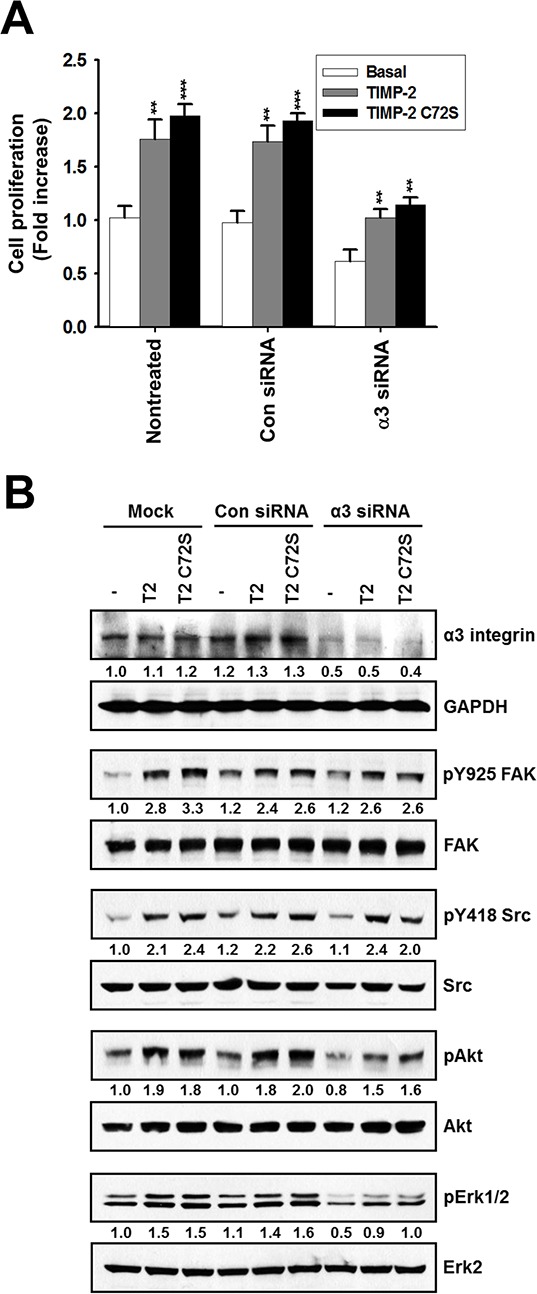
Effect of TIMP-2 or TIMP-2 C72S on growth-stimulatory activity in α3 integrin siRNA-transfected A549 cells **A.** A549 cells were transfected with either control siRNA or α3 integrin siRNA. After 24 hr, 250 pM TIMP-2 or TIMP-2 C72S was added to the transfected cells and non-treated cells. Following 24 hr, cell proliferation was measured by BrdU incorporation assay. Standard deviations were calculated from experiments performed in triplicate in three independent assays. Statistical significance is indicated. **p < 0.01; ***p < 0.001 when compared with untreated cells with TIMP-2 or TIMP-2 C72S. **B.** A549 cells were transfected with either control siRNA or α3 integrin siRNA using oligofectamine. After 24 hr, 250 pM TIMP-2 or TIMP-2 C72S was added to the transfected cells and the non-treated cells for 10 min. The cells were harvested and the cell lysates subjected to western blot analysis with antibodies that recognized α3 integrin, GAPDH, pY925 FAK, FAK, pY418 Src, Src, pS473 Akt, Akt, pERK1/2, and ERK2. Western blots shown are representative of three independent experiments. Relative band intensities were measured using Adobe photoshop software and integrated densities were normalized against the GAPDH, FAK, Src, Akt, or ERK2 loading control.

Our data led us to hypothesize that the mitogenic activity of TIMP-2 is mediated through another receptor besides MT1-MMP, a known cell-surface receptor for TIMP-2. Furthermore, MT1-MMP's catalytic activity is not inhibited by TIMP-2 C72S. Integrin α3β1, is another well-known receptor for TIMP-2. Therefore, we evaluated whether integrin α3β1 is involved in the mitogenic activity of TIMP-2. Cell proliferation assays and the phosphorylation status of c-Src, FAK, AKT, and ERK1/2 were evaluated in α3 integrin siRNA-transfected A549 cells and in control siRNA-transfected A549 cells [29]. We found that TIMP-2 and TIMP-2 C72S stimulated cell proliferation in both control and α3 integrin siRNA A549 cells (Figure 6A). Western blotting demonstrated that phosphorylation of c-Src, FAK, AKT, and ERK1/2 was increased by TIMP-2 and TIMP-2 C72S treatment in both control siRNA and α3 integrin siRNA A549 cells even though the basal phosphorylation levels of these proteins were reduced by α3 silencing in A549 cells (Figure 6B). These results suggest that TIMP-2 growth-stimulatory activity may be mediated through another receptor besides MT1-MMP and integrin α3β1.

### TIMP-2 expression is associated with a worse prognosis for pathological stage I lung adenocarcinoma

**Figure 7:**
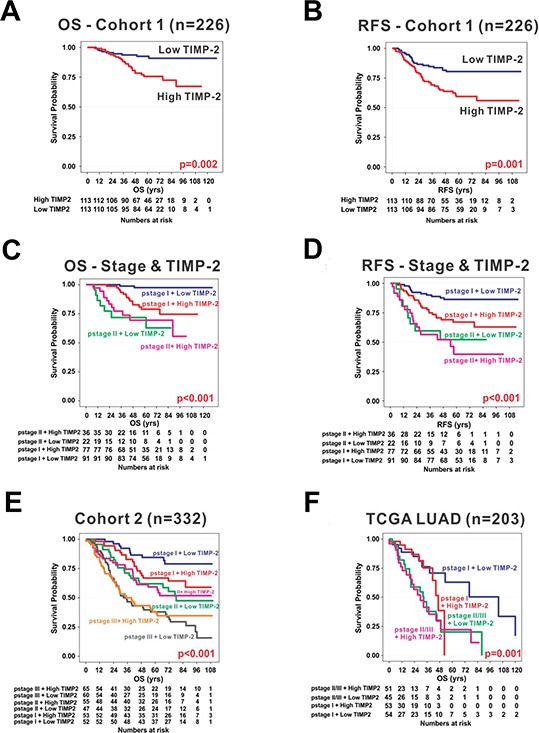
TIMP-2 expression and prognosis in lung adenocarcinoma Kaplan-Meier plots of overall survival (OS) **A.** and recurrence-free survival (RFS) **B.** in exploration cohort (*n* = 226) revealed that patients with high expression of TIMP-2 had worse prognosis when patients were dichotomized by median value of TIMP-2 expression. Distinct separation of OS **C.** and RFS **D.** survival curves by TIMP2 expression was confined to pathological stage I (pstage I) lung adenocarcinoma, but not pstage II. The finding that increased TIMP-2 expression is significantly associated with poor prognosis was consistent in another independent cohort (cohort 2; *n* = 332) **E.** and The Cancer Genome Atlas (TCGA LUAD) cohort (*n* = 203) **F.**

To evaluate the association between the growth-stimulatory activity of TIMP-2 and lung adenocarcinoma prognosis, we collected gene expression data from Gene Expression Omnibus (GEO) and TCGA. We divided the exploration cohort (*n* = 226; JNCC cohort, GSE31210) into two groups based on the median value of TIMP-2 expression (i.e., high and low TIMP-2 subgroups). Kaplan-Meier (KM) plots of overall survival (OS) and recurrence-free survival (RFS) revealed that patients with high TIMP-2 expression had worse prognosis (Figure 7A and 7B). Distinct separation of OS and RFS survival curves based on TIMP2 expression was confined to patients with pathological stage I (pstage I) lung adenocarcinoma (*p* < 0.001) (Figure 7C and 7D). Patients with pstage I lung adenocarcinoma and high TIMP2 expression had a similar survival curve to those patients with pstage II lung adenocarcinoma. When lung adenocarcinoma patients from another independent cohort (*n* = 332; MGH + ACC + Nagoya, GSE13213 and GSE11969) were divided into two groups according to TIMP-2 expression, Kaplan-Meier plots showed significant differences in OS for only pstage I (*p* < 0.001; Figure 7E). Survival in pstages II and III did not show any differences according to TIMP-2 expression. This finding is consistent with the TCGA lung adenocarcinoma cohort (*n* = 203) (Figure 7F) and suggests that TIMP-2 expression may promote oncogenic or growth-stimulatory changes only in pstage I lung adenocarcinoma.

### Integrated analysis of stage I TCGA lung adenocarcinoma

**Figure 8:**
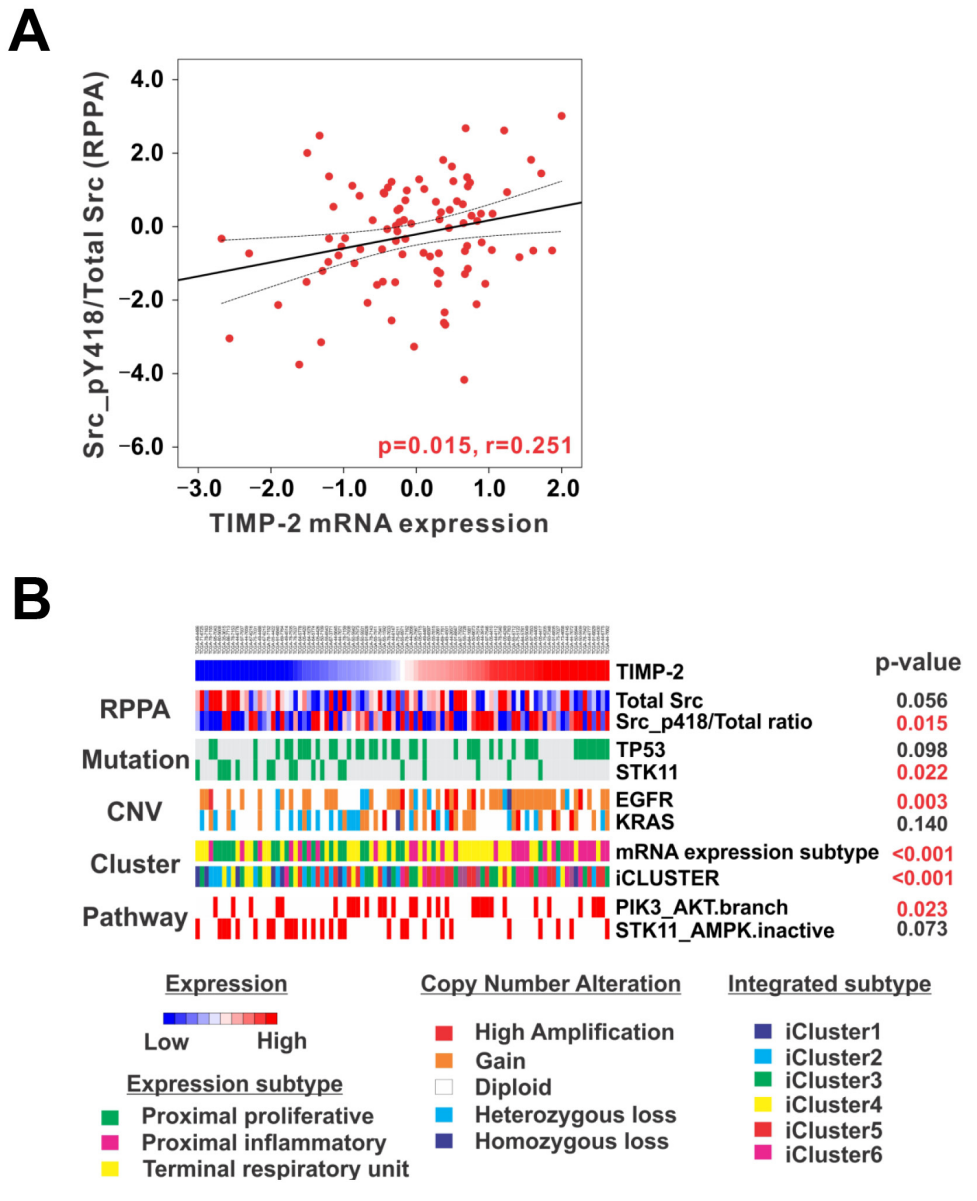
TIMP-2, Src phosphorylation, and PI3-kinase/AKT pathway alterations in stage I lung adenocarcinoma **A.** TIMP-2 mRNA expression showed a significantly positive correlation with Src phosphorylation at Y418 in stage I lung adenocarcinoma from The Cancer Genome Atlas Reverse Phase Protein Array (TCGA RPPA) database (*n* = 93). The r represents Pearson's correlation coefficient. **B.** 93 patients with stage I lung adenocarcinoma from TCGA database were sorted according to TIMP-2 mRNA expression to investigate its correlation with Src phosphorylation at Y418 and perform pathway analysis. High TIMP-2 expression TIMP-2 is associated with high rates of TP53 mutation, low rates of STK11 mutation, and copy number alteration of EGFR and KRAS. Furthermore, the proximal-inflammatory expression subtype is dominant in patients with high TIMP-2 expression. In contrast, the proximal-proliferative subtype is prevalent in patients with low TIMP-2 expression. Integrative Clusters 5 and 6 are more common in patients with high TIMP-2. Furthermore, the PIK3CA and AKT branch pathway is significantly activated in patients with high TIMP-2 expression, but SKT11-AMPK inactivation is related to low TIMP-2 expression.

To investigate the correlation between TIMP-2-induced Src phosphorylation and the alteration of driving genes, 93 patients with pstage I lung adenocarcinoma from TCGA database were sorted according to TIMP-2 mRNA expression. Reverse Phase Protein Assay (RPPA) data showed that Src phosphorylation at Y418 significantly increased when TIMP-2 was highly expressed (Figure 8A). High TIMP-2 expression was associated with high rates of TP53 mutation, low rates of STK11 (Serine/Threonine Kinase 11) mutation, and EGFR and KRAS copy number alterations (Figure 8B). In a paper about TCGA lung adenocarcinoma published by TCGA Research Network, RPPA, which provides proteomic and phosphoproteomic phenotypic evidence of pathway activity, revealed significant activation of mTOR and its effectors in a substantial fraction of the tumors [30]. Analysis of PIK3CA and STK11 mutations, STK11 protein levels, and AMPK and AKT phosphorylation led to the identification of three major mTOR patterns in lung adenocarcinoma: (1) tumors with minimal or basal mTOR pathway activation, (2) tumors showing higher mTOR activity accompanied by either STK11-inactivating mutation or combined low STK11 expression and low AMPK activation and (3) tumors showing high mTOR activity accompanied by either phosphorylated AKT activation, PIK3CA mutation, or both. Most interestingly, the PIK3CA and AKT branch pathway is significantly activated in patients with high TIMP-2 expression, but STK11-AMPK inactivation is related to low TIMP-2 expression (Figure 8B). These findings are consistent with our data suggesting that the growth-stimulatory activity of TIMP-2 is mediated through Src activation followed by the activation of PI 3-kinase and AKT, and patients with pstage I lung adenocarcinoma and high TIMP-2 expression have a poor prognosis.

Prior unsupervised analyses of lung adenocarcinoma gene expression have used varying nomenclature for transcriptional subtypes of the disease [31–34]. To coordinate naming of the transcriptional subtypes with the histopathological [35], anatomical, and mutational classifications of lung adenocarcinoma, TCGA Research Network proposed an updated nomenclature: the terminal respiratory unit (TRU, formerly bronchioid), the proximal-inflammatory (PI, formerly squamoid), and the proximal-proliferative (PP, formerly magnoid) transcriptional subtypes (Figure 7B) [30]. The PP subtype was enriched for inactivation of the STK11 tumor suppressor gene by chromosomal loss, inactivating mutation, and reduced gene expression. In contrast, the PI subtype was characterized by solid histopathology and co-mutation of NF1 and TP53. Finally, the TRU subtype harbored the majority of the EGFR-mutated tumors as well as the kinase fusion expressing tumors. These subtypes exhibited different mutation rates, transition frequencies, genomic ploidy profiles, and patterns of large-scale aberration. High TIMP-2 expression revealed more PI subtypes. In contrast, the PP subtype is prevalent in patients with low TIMP-2 expression. Furthermore, integrative clustering [36] of copy number, DNA methylation, and mRNA expression data found six clusters (Figure 7) [30]. This cluster membership was significantly associated with mutations in TP53, EGFR, and STK11 [30]. Integrative Clusters 5 and 6 are much more dominant in patients with high TIMP-2 expression. These findings imply that the activation of TIMP-2 in certain subtypes of pstage I lung adenocarcinoma may play a pivotal role in altering driver genes related to oncogenesis.

## DISCUSSION

In this study, we have shown that TIMP-2 growth-stimulatory activity is mediated by c-Src activation followed by activation of FAK, PI 3-kinase/AKT, and ERK1/2 through an MMP-independent mechanism in lung adenocarcinoma cells. In addition, by analyzing gene expression data from lung adenocarcinoma tissues, we showed that TIMP-2 expression is associated with a worse prognosis, especially in pstage I lung adenocarcinoma, from multiple cohorts.

In our experiments, TIMP-2 appeared to have the maximal effect on cell proliferation when used in pM concentrations with sparse cell numbers. The mitogenic activity of TIMP-2 decreased as cell-cell contact increased (data not shown). Although TIMP-2 has been shown to have A549 cell growth-stimulatory activity in previous reports, the effect of TIMP-2 on cell proliferation has remained controversial because other studies have shown that TIMP-2 inhibits cell proliferation in experiments that used the same types of cancer cells. TIMP-2 inhibited EGF-induced A549 cell proliferation [37]. *In vitro*, TIMP-2, which was overexpressed in A549 cells using a retroviral delivery system, did not alter basal cell proliferation rates. *In vivo*, TIMP-2 A549 xenografts exhibited reduced tumor growth by inhibiting tumor angiogenesis and inducing tumor cell apoptosis [38]. Taken together, these studies suggest that TIMP-2 increases tumor cell growth before the onset of angiogenesis during the initial period of tumor development. Following this initial period, TIMP-2 expression and activity may, in turn, reduce tumor growth by inhibiting tumor angiogenesis and inducing tumor cell apoptosis. There is a published study that supports this hypothesis. Induction of TIMP-1 expression in an EBV-negative Burkitt's lymphoma cell line results in a biphasic tumor growth in nude mice. The initial period of tumor growth is enhanced by TIMP-1 expression and this period was followed by tumor necrosis and tumor regression due to TIMP-1 inhibition of an angiogenic response within the developing tumor mass [39]. Also, the potential for TIMP-2 to either promote or inhibit cell growth could be affected by the concentration of TIMP-2 and the presence or absence of growth factors such as EGF or PDGF in the tumor microenvironment. Further investigation regarding the biphasic effect of TIMP-2 is warranted.

Reports have shown that TIMP-2 stimulates cell proliferation in HT-1080 cells and MG-63 cells via a cAMP/PKA-dependent mechanism [14, 20]. In our study, H89, a PKA inhibitor, reduced TIMP-2-induced cell proliferation; however, inhibition of adenylate cyclase by SQ22536 or use of PKAα cata siRNA did not block the growth - stimulatory effect of TIMP-2 in A549 cells. H89 inhibits other signaling molecules such as casein kinase I, MLCK, PKC, and ROCK-II. We found that inhibition of PKC, one of the targets of H89, considerably reduced TIMP-2-stimulated cell proliferation. Our results suggest that TIMP-2 growth-stimulatory activity may involve casein kinase I, MLCK, ROCK-II, and/or PKC via a cAMP/PKA independent mechanism in A549 cells.

Several studies have shown that the activation of c-Src is necessary for the mitogenic responses of growth factors, such as PDGF or EGF, which are implicated in tumorigenesis [40]. In this study, we have shown that the activation of c-Src kinase plays a key role in the growth-stimulatory activity of TIMP-2 in A549 cells. We did not detect changes in Src phosphorylation at Y529; however, TIMP-2 treatment increased Src phosphorylation at Y418. It has been demonstrated that 90–95% of Src is phosphorylated at Y529 under basal conditions *in vivo* [41]. It seems that most of Src is phosphorylated at Y529 under basal condition in A549 cells. Only a small proportion of total Src could be dephosphorylated at Y529 and concomitantly phosphorylated at Y418 by TIMP-2. Most of the Src protein in cells remains phosphorylated at Y529. We believe this is why we could not detect changes in Y529 phosphorylation status on c-Src.

Our data also demonstrated that a PI3-kinase inhibitor strongly reduced TIMP-2 induced phosphorylation of ERK1/2. This result suggests that PI3-kinase may be upstream of ERK1/2. Ras proteins are able to activate PI3-kinase, and PI3-kinase has been shown do be involved in the Ras/Raf/MEK/ERK1/2 pathway. Activation of both ERK2 and Ras by low concentrations of EGF is suppressed by PI3-kinase inhibitors [42]. The activation of Ras by low, but mitogenic concentrations of EGF is dependent on basal PI 3-kinase activity. TIMP-2 may activate Ras via the same mechanism. However, further experiments using dominant negative proteins will be needed to define the exact signaling pathways (PI3-kinase, AKT, or ERK1/2) that are effectors of c-Src activation.

MT1-MMP and α3β1 integrin are known to be cell-surface receptors for TIMP-2 [29, 43]. TIMP-2 binds to the surface of human microvascular endothelial cells via interaction with the α3β1 integrin and this interaction suppresses VEGF-A- or FGF-2-induced cell proliferation *in vitro* and angiogenesis and tumorigenesis *in vivo* [29]. We showed that TIMP-2 significantly increased the phosphorylation of c-Src, FAK, AKT, and ERK1/2 in α3 integrin siRNA A549 cells when compared with control siRNA A549 cells, suggesting that there is another TIMP-2 receptor besides α3β1 integrin and MT1-MMP. Future studies will be needed to identify the receptor that mediates the mitogenic activity of TIMP-2.

The American Joint Committee on Cancer staging system is currently used to guide treatment decisions and is the best predictor of prognosis for patients with NSCLC. While postoperative adjuvant chemotherapy is the standard care among patients with stage II to IIIA NSCLC who have undergone complete resections, surgery remains as the only recommended guideline for treatment of stage I NSCLC. Of those patients with stage IA and IB NSCLC who are candidates for surgical resection, the 5-year OS is approximately 73% and 58%, respectively [44, 45]. The recurrence rate in stage I patients who underwent surgical resection ranges from 13–41% [46, 47]. Thus, postoperative recurrence is a major obstacle to prolonged survival in early stage NSCLC, and considerable differences exist among patients with the same pathological stage. This indicates that NSCLC is a very heterogeneous cancer even in the earliest stage, and this underlying heterogeneity is not well-reflected in the current staging system. A small fraction of NSCLC patients have underlying EGFR mutations or EML4-ALK fusions that are associated with relatively high response rates to targeted molecular therapies [48–50]. However, for the majority of adenocarcinoma patients, we do not yet have any validated biomarkers to predict overall outcome or to guide treatment selection. Genetic markers that could identify those at high risk for recurrence are essential for developing an effective clinical practice strategy, as patients with a poor prognosis could undergo more advanced treatment regimens, such as the use of targeted agents. The robustness of TIMP-2 growth-stimulatory gene expression was validated in two independent cohorts with a total of 861 patients. Since current staging systems and biomarkers are limited in their ability to assess risk of recurrence and benefit from adjuvant chemotherapy in early stage lung adenocarcinoma, our mechanistic investigation of TIMP-2 growth-stimulatory activity may represent a tool that could help further refine treatment decisions based on the tumors' molecular profiles. Using integrated analysis, we identified new pathway-dependent prognostic subgroups of lung adenocarcinoma that show significant differences in patient survival, especially in pstage I lung adenocarcinoma. This study provides new knowledge by illuminating modes of genomic alteration, highlighting previously unappreciated TIMP-2-related genes, and enabling further refinement in sub-classification for improved personalization of treatment for this deadly disease. Thus, our results, if confirmed in prospective studies, may improve patient care by providing more practical guidance for treatment.

## MATERIALS AND METHODS

### Reagents and antibodies

Epidermal growth factor and insulin were purchased from Sigma-Aldrich (St Louis, MO). Signaling inhibitors including SQ22536, H89, PD98059, LY294002, NF-κB inhibitor, PP3, PP2, and FAK inhibitor I were obtained from EMD Millipore Bioscience (Billerica, MA). PKC inhibitor (Gö 6976) was purchased from A.G. Scientific, Inc. (San Diego, CA). The α3-integrin siRNA, PKAα cat siRNA, c-Src siRNA, and control siRNA were purchased from Santa Cruz Biotechnology, Inc. (Santa Cruz, CA). Anti-pY418 Src, anti-pY529 Src, anti-Src, anti-pY925 FAK, anti-pS473 Akt, and anti-Akt antibodies were obtained from Bioworld (St Louis Park, MN). Anti-FAK, anti-pErk1/2, anti-Erk2, anti-PKAα, and anti-α3 integrin antibodies, and horseradish peroxidase-conjugated secondary antibodies were purchased from Santa Cruz Biotechnology, Inc. Enhanced chemiluminescent detection kit was purchased from GE Healthcare Bio-Sciences (Pittsburgh, PA). Anti-GAPDH was purchased from R&D Systems, Inc. (Minneapolis, MN).

### Cell culture and establishment of stable cell lines

A549 cells were obtained from the American Type Culture Collection and SK-LU-1, HCC-827, and A427 cells were purchased from the Korean Cell Line Bank. NCI-H2009 cells were kindly provided from Dr. Jong Ho Park (Korea Institute of Radiological and Medical Sciences, Korea). A549, SK-LU-1, A427, and NCI-H2009 cells were cultured in Dulbecco's modified Eagle's medium (Life technologies, Grand Island, NY) and HCC-827 cells were cultured in RPMI-1640 medium (Life technologies, Grand Island, NY). All media was supplemented with 5% FBS, 100 unit/ml penicillin, and 0.1 mg/ml streptomycin at 37°C. Stable cell lines expressing only vector or kinase-dead (K297R) Src were generated by transfection of A549 pLNCX or pLNCX-kinase-dead (K297R) Src, which was kindly provided by Dr. Youn Soo Kim (Inje University, Korea), into A549 cells by the calcium phosphate method and selection with 1 mg/mL neomycin.

### Western blot analysis

Cells were lysed in lysis buffer, including protease inhibitor cocktail (Sigma) and phosphate inhibitor cocktail I and II (EMD Millipore Bioscience, Billerica, MA), on ice for 30 min. Protein concentrations were determined using the microBCA assay (Pierce, Rockford, IL). For Western blot analysis, proteins lysates (60 μg) were resolved by sodium dodecyl sulfate polyacrylamide gel electrophoresis and transferred to nitrocellulose membranes (GE healthcare Bio-Sciences, Pittsburgh, PA). Immunoreactive proteins were visualized with anti-pY418 Src, anti-pY529 Src, anti-Src, anti-pY925 FAK, anti-FAK, anti-pS473Akt, anti-Akt, anti-pErk1/2, anti-Erk2, anti-PKAα, anti-α3 integrin, and anti-GAPDH primary antibodies, horseradish peroxidase-conjugated secondary antibody, and an enhanced chemiluminescent detection kit (GE healthcare Bio-Sciences, Pittsburgh, PA).

### Cell proliferation assay

Cell proliferation was measured using a BrdU cell proliferation assay kit (Cell Signaling Technology, Inc., Danvers, MA). Subconfluent cells were starved in serum-free DMEM for 24 hr. TIMP-2 or TIMP-2 C72S was added to the cells at the indicated concentrations. In the experiments using signaling inhibitors, cells were starved in serum-free DMEM for 24 hr and were pretreated with SQ22536 (100 μM), H89 (5 μM), PD98059 (10 μM), LY294002 (1 μM), NF-κB inhibitor (1 μM), PP3 (10 μM), PP2 (10 μM), FAK inhibitor I (10 μM), or PKC inhibitor (100 nM) for 30 min. Cells were subsequently treated with 250 pM of either TIMP-2 or TIMP-2 C72S and further incubated for 24 hr. Finally, 10 μM BrdU was added to the plate and cells were incubated with BrdU containing medium for 4 hr. BrdU incorporation was measured using the BrdU cell proliferation assay kit according to the manufacturer's instructions.

### Knockdown of PKAα catalytic subunit, c-Src, and α3 integrin in A549 cells

A549 cells were transfected with various siRNAs [PKA**α** cat siRNA (sc-36240), 80 nM; α3-integrin siRNA (sc-35684), 50 nM; c-Src siRNA (sc-29228), 50 nM; control siRNA (sc-37007), 50 nM] (Santa Cruz Biotechnology) using Oligofectamine (Life technologies, Grand Island, NY) and Opti-MEM reduced serum media (Life technologies, Grand Island, NY). Reduced protein expression of PKAα catalytic subunit, α3-integrin, and Src were confirmed via western blot after 48 hr of transfection.

### Preparation of TIMP-2 or TIMP-2 C72S protein

Site-directed mutagenesis was used to introduce mutations into the human TIMP-2 cDNA cloned into the *Hin*dIII and *Eco*RI sites of the pcDNA3 vector (Life Technologies, Grand Island, NY) [51]. The TIMP-2 C72S mutant was generated using the MutaDirect site-directed mutagenesis kit (Intron biotechnology, Sungnam, Kyungki-do). The mutagenic primers are listed below (the mutated amino acid codon is underlined): C72S forward (5′-CTCCTCGGCAGTGAGTGGGGTCTCG-CTGGAC-3′) and C72S reverse (5′-GTCCAGCGAGACCCCACTC ACTGCCGAG-GAG-3′). The resulting construct was sequenced to verify the mutagenesis. HEK 293 cell lines stably expressing TIMP-2 and TIMP-2 C72S were generated by calcium phosphate transfection of HEK 293 cells with pcDNA3-TIMP-2 and pcDNA3-TIMP-2 C72S followed by selection with 1,200 μg/mL G418 [52, 53]. Purification of TIMP-2 or TIMP-2 C72S was followed by a method described (Supplementary Figure S1) [54]. The catalytic and hinge domain (Tyr112-Ile318) of the human membrane type-1 matrix metalloproteinase (cMT1-MMP) were expressed in *E. coli* and refolded actively according to a previously described method [55]. The recombinant human proMMP-2 was expressed in Sf9 cells with infection of the 72Gel baculovirus and purified with gelatin-agarose column chromatography as described [53]. The catalytic activity of either cMT1-MMP or the APMA-activated MMP-2 was measured by fluorogenic peptide cleavage assay in the presence of various concentrations of TIMP-2 or TIMP-2 C72S [53].

### Src kinase assay

For the Src tyrosine kinase assay, 500 μg of protein lysate were incubated with 1 μg of anti-Src antibody for 5 hr followed by incubation wtih 20 μl of protein A/G beads (1:1 slurry) for 1 hr at 4°C. Src tyrosine kinase activity in 1/10 of the immunoprecipitated protein was assayed using a c-Src kinase assay kit (The CycLex Research Product).

### Gene expression data and patients cohorts

Gene expression and clinical data from the National Cancer Center Research Institute in Japan (JNCC cohort, *n* = 226) were obtained from the National Center for Biotechnology Information (NCBI) Gene Expression Omnibus (GEO) database (http://www.ncbi.nlm.nih.gov/geo, accession number GSE31210) and used [56]. For validation, gene expression and clinical data from Massachusetts General Hospital (MGH cohort, *n* = 125) were obtained from the public website of the Broad Institute (http://www.broadinstitute.org/mpr/lung) [34] and data from the Aichi Cancer Center (ACC cohort, GSE13213, *n* = 117) and Nagoya (GSE11969) were obtained from the National Center for Biotechnology Information (NCBI) Gene Expression Omnibus (GEO) database (http://www.ncbi.nlm.nih.gov/geo, accession number). These three cohorts were merged and used as the validation set (*n* = 332). Moreover, lung adenocarcinoma data from TCGA database was leveraged for validation and integrated analysis. All patients had undergone surgical resection as their primary treatment. Although OS and RFS were available for JNCC cohorts, only OS data were available for the other cohorts (MGH, ACC, Nagoya, and TCGA). Therefore, we used OS as an end point.

### Statistical analysis of microarray data

Biometric Research Branch (BRB)-Array Tools were used for statistical analysis of the gene expression data [57], and all other statistical analyses were performed in the R language environment (http://www.r-project.org). Except for data from the ACC and Nagoya cohorts, all gene expression data were generated using the Affymetrix (Santa Clara, CA) platform (U95A for the MGH cohort and U133 plus 2.0 for the JNCC cohorts). Raw data from the Affymetrix platform were downloaded from public databases and normalized using a robust multi-array averaging method [58]. Data from the ACC and Nagoya cohorts were generated using the Agilent whole-genome microarray platform, and pre-normalized data were downloaded and used for analysis. We divided the discovery cohort into two groups by the median value of TIMP-2 expression (high and low TIMP-2 subgroups). Cluster analysis was performed with Cluster and Treeview [59]. When applied to the independent validation sets, prognostic significance was estimated by evaluating the differences between Kaplan-Meier plots and log-rank tests between the two subgroups of patients. Statistical analyses were performed using a Student's *t*-test and were based on at least three different experiments. Statistical significance is indicated (*p < 0.05; **p < 0.01; ***p < 0.001) and all tests were two-tailed.

### Integrative analysis from TCGA dataset

We obtained mRNA sequencing data of lung adenocarcinoma (LUAD) from TCGA portal (https://tcga-data.nci.nih.gov/tcga/). Normalized reads per kilobase per million values were transformed logarithmically and centralized by subtracting the mean of each channel and further normalized to equalize the variance. We leveraged the LUAD data (*n* = 93), including mRNA, somatic mutation, DNA copy number alteration, and clinical data, from TCGA portal and the cBioPortal for Cancer Genomics (http://www.cbioportal.org/public-portal/index.do). To visualize genomic alterations in multiple genes, we used OncoPrint generated by cBioPortal for Cancer Genomics. Then, we analyzed the frequency of genetic alteration in major genes [60].

## SUPPLEMENTARY FIGURE


