# Supplementary material for: Growth-stimulatory activity of TIMP-2 is mediated through c-Src activation followed by activation of FAK, PI3-kinase/AKT, and ERK1/2 independent of MMP inhibition in lung adenocarcinoma cells
Source: Oncotarget. 2015 Nov 7;6(40):42905–22. doi: 10.18632/oncotarget.5466 (PMC4767480; doi:10.18632/oncotarget.5466)
Supplement: Supplementary file 1 [file oncotarget-06-42905-s001.pdf]

SUPPLEMENTARY FIGURE

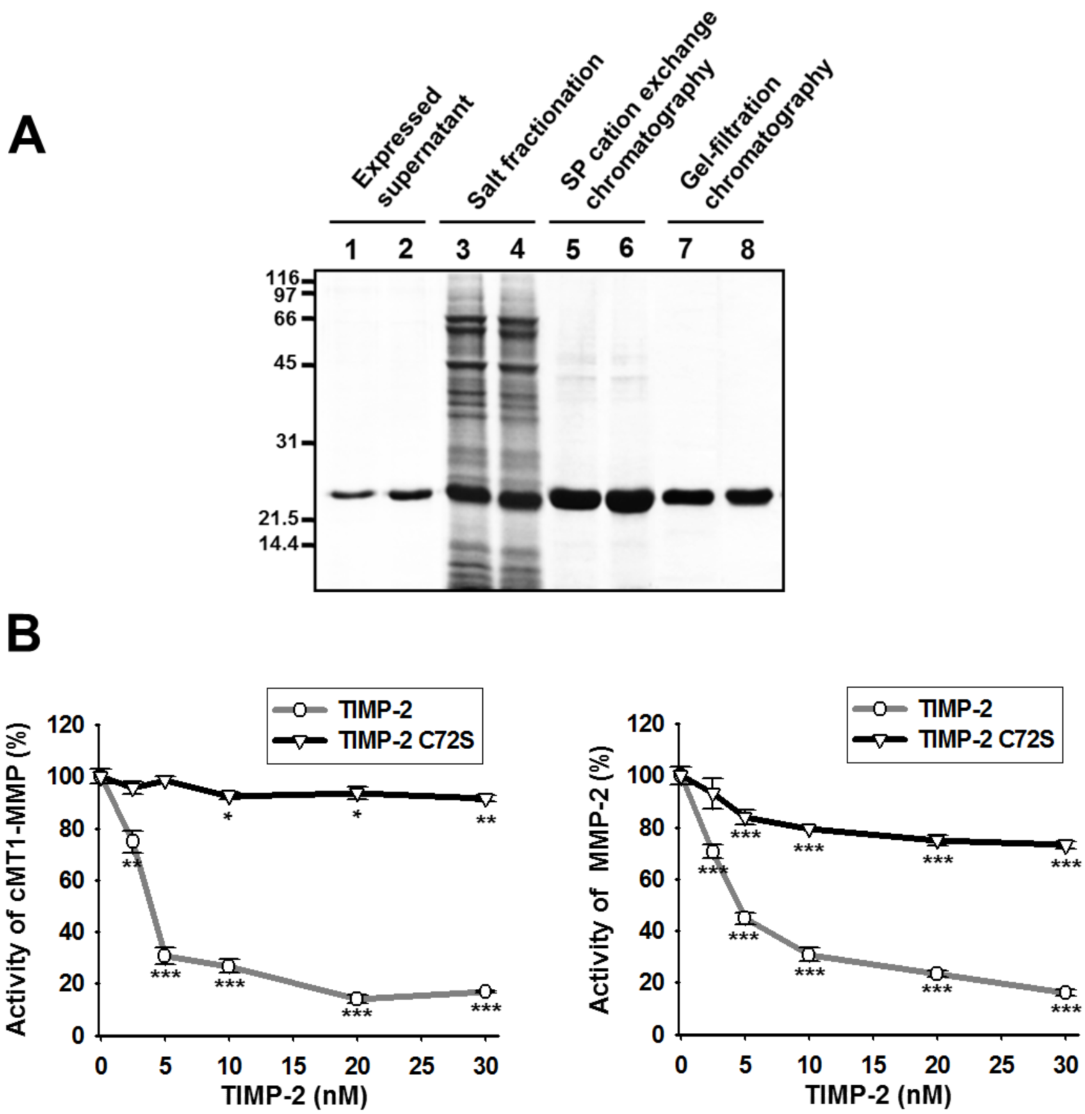

Supplementary Figure S1: Purification and characterization of TIMP-2 and TIMP-2 C72S protein. A. SDS-PAGE of protein samples. A small amount of protein of the indicated purification steps of TIMP-2 and TIMP-2 C72S was loaded into lanes 1, 3, 5, and 7 and lane 2, 4, 6, and 8, respectively. **B.** The catalytic activity of either cMT1-MMP or the activated MMP-2 was measured by fluorogenic peptide cleavage assay, in the presence of the indicated amounts of TIMP-2 or TIMP-2 C72S. The catalytic activity (%) of each MMP in the presence of TIMP-2 or TIMP-2 C72S divided by the catalytic activity in the absence of TIMP-2 or TIMP-2 C72S.
